# Accumulations of Heavy Metals in Roadside Soils Close to Zhaling, Eling and Nam Co Lakes in the Tibetan Plateau

**DOI:** 10.3390/ijerph10062384

**Published:** 2013-06-07

**Authors:** Xuedong Yan, Fan Zhang, Dan Gao, Chen Zeng, Wang Xiang, Man Zhang

**Affiliations:** 1 MOE Key Laboratory for Urban Transportation Complex Systems Theory and Technology, Beijing Jiaotong University, Beijing 100044, China; E-Mails: xdyan@bjtu.edu.cn (X.Y.); 11121095@bjtu.edu.cn (D.G.); 12114242@bjtu.edu.cn (W.X.); 10125479@bjtu.edu.cn (M.Z.); 2 Key Laboratory of Tibetan Environment Changes and Land Surface Processes, Institute of Tibetan Plateau Research, Chinese Academy of Sciences, Beijing 100101, China; E-Mail: zengchen@itpcas.ac.cn

**Keywords:** Tibetan Plateau, heavy metals, traffic activities, hierarchical tree-based regression, potential ecological risk

## Abstract

Concentrations of four typical heavy metals (Cu; Zn; Cd and Pb) in roadside soils close to three lakes in the Tibetan Plateau were investigated in this study. The hierarchical tree-based regression method was applied to classify concentrations of the heavy metals and analyze their potential influencing factors. It was found that the Tibetan Plateau meadow soils with higher content of sand lead to higher concentrations of Cu; Zn and Pb. The concentrations of Cd and Pb increase with road traffic volume; and for the road segments with higher traffic volume; the Cd and Pb concentrations significantly decrease with the roadside distance. Additionally; the concentrations of Zn and Pb increase as the altitude of sampling site increases. Furthermore; the Hakanson potential ecological risk index method was used to assess the contamination degree of the heavy metals for the study regions. The results show that accumulations of Cu; Zn and Pb in roadside soils remain an unpolluted level at all sites. However; the Cd indices in the regions with higher traffic volume have reached a strong potential ecological risk level; and some spots with peak concentrations have even been severely polluted due to traffic activities.

## 1. Introduction

The Tibetan Plateau and surrounding mountains are named the Third Pole of the World due to its high altitude (over 4,000 m), large area of more than 5 million km^2^, unique geographical position and special climate environment effect [1,2]. Compared to all the other giant geomorphic units in the World, the Tibetan Plateau’s latitude is the lowest, area is largest, and geological formation time is the latest. The changes of the Tibetan Plateau environment strongly influence its alpine ecosystem and it responds sensitively to global climate change. The environment of the Tibetan Plateau is relatively less affected by anthropogenic interferences because of its natural agricultural structure, low population density and extremely sparse industrial activities owing to current Chinese environmental protection policy. However, traffic contaminants are one of the increased concerns in the Tibetan Plateau environment. In recent years, with the quick development of Chinese economy, the numbers of motor vehicles and tourists in the Tibetan Plateau have been increasing rapidly. Road traffic activities are the major source of heavy metal emission to roadside soils [3] and the highest heavy metal concentrations can be found in the zones with heavy traffic [4]. Therefore, studies on heavy metal accumulation caused by road traffic are of great significance to the region’s environmental protection and human health safety.

Numerous previous studies have investigated the influencing mechanism of vehicle emissions on concentrations of heavy metals in roadside soils [5,6]. Usually, the elements used to assess traffic contamination are Cu, Zn, Cd and Pb [7,8]. The concentrations of heavy metals decrease with increasing roadside distance [9,10]. Pollutants containing heavy metals enter soils through various approaches [11], including colloid chemistry processes, biological processes and physical processes, *etc.* The metals in soil may be washed away by rain and road runoff and eventually get into adjacent river systems [12]. Heavy metals, no matter whether in soil or water, may accumulate in plants, animals, or human bodies and thus constitute a threat to public health [13]. Even extremely low doses of heavy metals may cause serious diseases in human beings. For example, Cd is the most hazardous heavy metal to human health [14] and excessive Cd exposure may give rise to renal, pulmonary, hepatic, skeletal and reproductive effects, as well as cancer [13]. Pb poisoning can cause neurologic and behavioral disorders and it can affect blood Pb levels [15]. Cu and Zn are essential elements in the human body, but excess doses of them will cause diseases. Thus, the heavy metal contamination in the Tibetan Plateau should not be ignored. 

The metal contamination of roadside soils may involve adjacent aquatic systems because the pollutants in roadside soils can be transported into nearby lakes through rainfall and surface runoff. Some studies have been conducted to investigate the effects of lakeshore reclamation on water quality of lakes or adjacent surface water contamination [16]. Dynamically monitoring and assessing heavy metals contamination of roadside soils in the regions close to plateau lakes has been particularly concerned for wetland ecosystem health and regional ecological security [17]. The heavy metals in aquatic systems may accumulate in aquatic organism [18], and the consumption of these aquatic food enriched with toxic heavy metals may cause human health problems through food chain [19]. However, little research paid attention to the evaluation of heavy metals accumulation in roadside soils nearby lakes under the special environment of the Tibetan Plateau.

Due to rapidly increasing tourism and transportation activities in the Tibetan Plateau, heavy metals accumulations in roadside soils adjacent to three lakes and in adjacent regions were evaluated in this study. The roadside soils nearby Zhaling and Eling Lakes and Nam Co Lake in the Tibetan Plateau were selected as sampling objects. The Tibetan Plateau involves Qinghai Province and the Tibet Autonomous Region in China. Zhaling and Eling Lakse in Qinghai Province are the source of the Yellow River, the second largest river in China. The two lakes belong to Chinese national nature reserves and were listed in the International Important Wetland Directory in 2005. In the Tibet Autonomous Region, Nam Co Lake is the biggest saltwater lake. This paper aims to: (1) measure the heavy metal content in roadside soils near plateau lakes and in adjacent regions; (2) analyze the main influencing factors associated with the heavy metal concentrations and distributions; and (3) assess the local heavy metal contamination extent due to vehicle emission.

## 2. Materials and Methods

### 2.1. Site Description

**Figure 1:**
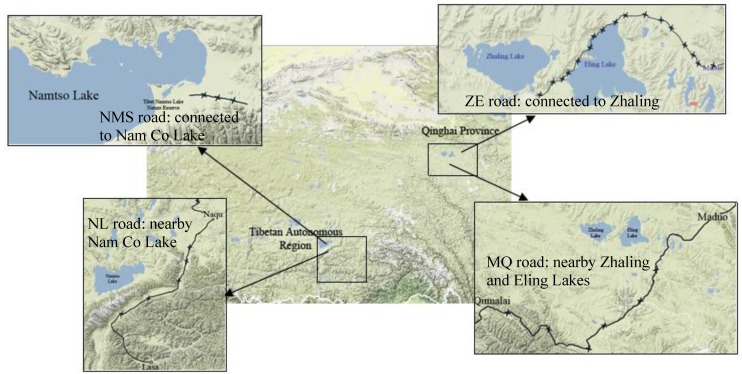
Locations of four road segments and sampling sites.

In this study, 96 soil samples were collected along four road segments in the Tibetan Plateau. They are named as ZE road, MQ road, NMS road and NL road, respectively. As shown in Figure 1, the ZE road is connected to both Zhaling and Eling Lakes, and the MQ road is a segment close to the two lakes in Qinghai Province; the NMS road goes along Nam Co Lake, and the NL road is a segment of national highway #109, close to the Nam Co Lake in the Tibet Autonomous Region. The ZE road is located at an area with an altitude over 4,100 m. The MQ road’s altitude ranges from 4,100 m to 4,700 m. In order to avoid the complex roadside terrain, only nine samples were collected along the NMS road segment along the Nam Co Lake. The altitude of Nam Co Lake is about 4,700 m. NL road is a two-way road and its length is 546 km and its width is 7 m. The altitude in this area is in the range of 4,200 m to 4,700 m. Most of the soils along the four road segments are meadow soils, which have sufficient moisture, and organic matter and the humus layer is thick. However, the content of sands in the roadside soils varies from site to site. The order of traffic volume of the road segments is ZE < NMS < MQ < NL. The NL road segment of national highway #109 is the first choice for most people traveling in Tibet. MQ road is a part of national highway #214 and there are abundant tourism resources along the road. In recent years, more and more visitors to the Nam Co Lake have resulted in a relatively high traffic density on the NMS road compared to the ZE road.

### 2.2. Soil Sampling and Processing

A total of 96 topsoil samples were collected under dry weather conditions along the four road segments during July 2011. In order to avoid the complex roadside terrain, 32 sampling sites were identified along the road segments in the study regions. For each sampling site, three topsoil (0–5 cm) samples were collected according to 0 m, 10 m and 50 m roadside distances. For each sample, 8–10 topsoil sub-samples were taken in an “S-shape” pattern in a 10 m × 2 m plot and evenly mixed. In order to analyze the potential influencing factors associated with the heavy metal concentrations and distributions in roadside soils, during the data collection, DIST, SOIL, PRE, ALT, ROAD, VOLUME and REGION are named as the independent variables related to the soil samples. The variables’ descriptions and classifications as well as the sample distribution are shown in Table 1.

**Table 1:** Description of independent variables related to the soil samples.

Variable	Description	Class	Number of samples	Percentage (%)
DIST	Roadside distance	0 m: Road edge	32	33.3
		10 m: 10 m distance from road edge	32	33.3
		50 m: 50 m distance from road edge	32	33.3
SOIL	Roadside soil type	Meadow: Pure meadow soil	69	71.9
		Mea-ls: Meadow soil with low content of sand	9	9.3
		Mea-hs: Meadow soil with high content of sand	18	18.8
PRE	Air Pressure measured at sampling sites	560 Pa–570 Pa	3	3.1
		570 Pa–580 Pa	9	9.4
		580 Pa–590 Pa	12	12.5
		590 Pa–600 Pa	9	9.4
		600 Pa–610 Pa	6	6.3
		610 Pa–620 Pa	57	59.4
ALT	Altitude of sampling sites	4,100 m–4,200 m	51	53.1
		4,200 m–4,300 m	3	3.1
		4,300 m–4,400 m	3	3.1
		4,400 m–4,500 m	6	6.3
		4,500 m–4,600 m	9	9.4
		4,600 m–4,700 m	6	6.3
		4,700 m–4,800 m	12	12.5
		4,800 m–4,900 m	6	6.3
ROAD	The road pavement surface type	Asphalt	63	65.6
		Gravel	33	34.4
VOLUME	The level of road segment traffic volume	Low	60	62.5
		Middle	18	18.8
		High	18	18.8
REGION	The sampling region in terms of road segment	ZE: connected to Zhaling Lake and Eling Lake	51	53.1
		MQ: nearby Zhaling Lake and Eling Lake	18	18.8
		NMS: connected to Nam Co Lake	9	9.4
		NL: nearby Nam Co Lake	18	18.8

It should be noted that the roadside soil type was categorized into three classes according to the researchers’ onsite subjective evaluation on the content of sand in meadow soil. Air pressure and altitude were measured at each sampling site using a GPS device.

The samples were dried at room temperature and then ground with an agate mortar until all of the soils passed the 100 mesh nylon sieve. Soil (0.3 ± 0.0001 g) was digested in a microwave (CEM Mars X microwave digestion oven, Matthews, NC, USA) using a mixture of acids (6 mL HNO_3_, 3 mL HCL and 0.25 mL H_2_O_2_) in a digestion tube. The digestion process is shown in Table 2. Then, the digestion liquid was analyzed by the Inductively Coupled Plasma-Mass Spectrometry (ICP-MS, Thermo X Series 2, Thermo Fisher Scientific Inc., Waltham, MA, USA). The AVG-2 and GSS-8 standard materials were used as quality control in the experimental process, and the relative standard deviation was less than 10%.

**Table 2:** The microwave digestion process of soil samples.

Time (min)	Temperature (°C)	Hold (min)
5	120	5
5	160	5
5	190	40

### 2.3. Data Analysis Methods

In this study, the hierarchical tree-based regression method was used to classify concentrations of heavy metals and analyze the potential influencing factors. The Hakanson potential ecological risk index method was used to assess the contamination degrees of heavy metals for the study regions.

#### 2.3.1. Hierarchical Tree-Based Regression

In previous studies on explaining heavy metal concentration patterns, the statistical methods such as analysis of variance and hierarchical clustering have been employed. However, no one has applied the hierarchical tree-based regression (HTBR) [20] to statistically explore the heavy metal concentration data. HTBR classifies observations by recursively partitioning the predictor space. Due to its nonparametric nature and easy interpretation, HTBR is very popular in various fields. In tree-structured representations, the entire dataset is represented by a root node. When a split is made, child nodes that correspond to partitioned subsets are formed. If a node is not to be split any further, it is called a terminal node that is associated with a group membership; otherwise, it is an internal node. The tree is constructed following a set of decision rules applied sequentially. Each decision rule is used to form branches (*i.e.*, splitting) connecting the root node to the terminal node at a certain level of the tree. Based on the decision rules, the HTBR model’s explanation is straightforward for both analysis and predictive purposes.

The HTBR procedure creates a tree-based classification model using the Classification and Regression Trees (CART), Chi-squared Automatic Interaction Detection (CHAID), or Quick, Unbiased, Efficient, Statistical Tree (QUEST) algorithms. Among the three algorithms, QUEST can be used only for the nominal dependent variable and CART will always yield binary trees, which sometimes cannot be summarized as efficiently for interpretation and/or presentation [20]. Therefore, the CHAID algorithm is applied for this study because it allows multi-way splits of a node and is not limited to nominal dependent variable. The CHAID algorithm, originally proposed by Kass [21] and further developed by Magidson [22], automatically determines the most significant spilt criteria based on Chi-square tests to measure the association between dependent variable and independent variables. The procedure consists of three steps: first preparing predictors, then merging categories, and finally selecting the split variable. The process continues until no further splits can be performed (given the alpha-to-merge and alpha-to-split values).

In this study, the target variables are the concentrations of Cu, Zn, Cd and Pb. The independent factors in this study include DIST, SOIL, PRE, ALT, ROAD, VOLUME and REGION. The nominal input variables’ descriptions and correlative statistics are listed in Table 1. We used the CHAID growing method to build tree models. The HTBR analyses in this study were carried out using the SPSS software package (version 16.0; SPSS Inc., Chicago, IL, USA). The specifications for tree construction include: the maximum tree depth was set to three levels; the minimum number of cases for parent nodes was set to 10 and the minimum number of cases for child nodes was set to five; the significance values for both splitting nodes and merging categories are set to 0.05. In order to select the best tree size, the 10-fold cross-validation method was used to assess how well each subtree generalizes to a larger population and hence determine the best final tree model.

#### 2.3.2. Hakanson Potential Ecological Risk Index Method

The method of Hakanson potential ecological risk index was used to assess the degree of the heavy metal pollution and its potential ecological harm [23]. The index is calculated by Equation (1):

*E^i^_r_* = *T^i^_r_* × C*^i^_f_*;C*^i^_f_* = C*^i^_s_*/C*^i^_n_* (1)

where *E*^*i*^_*r*_ is the potential ecological risk index for heavy metal *i*; *T^i^*_*r*_ is the toxic-response factor of a single pollution element； *C^i^_f_* is the pollution coefficient of heavy metal *i*; *C^i^_s_* is the measured concentration of heavy metal *i* in the soil (mg·kg^−1^); *C^i^_n_* is the background value of heavy metal *i* in deposits.

The ecological risk degree is divided into five levels: *E^i^*_*r*_ < 40 represents a low risk level; 40 ≤ *E^i^*_*r*_ < 80 represents a moderate risk level; 80 ≤ *E^i^*_*r*_ <160 represents a strong risk level; 160 ≤ *E^i^*_*r*_ <320 represents a very strong risk level; *E^i^*_*r*_ ≥ 320 represents an extremely strong risk level [24]. The background values of Cu, Zn, Cd and Pb are 22.2, 80.3, 0.137 and 20.9 mg/kg respectively in Qinghai Province, and they are 21.9, 74, 0.081 and 29.1 mg/kg in the Tibet Autonomous Region [25]. The toxic response parameters of Cu, Zn, Cd and Pb are 5, 1, 30 and 5, commonly adopted in previous studies [26].

## 3. Results and Discussion

### 3.1. Descriptive Statistical Analyses of Heavy Metal Concentrations

The basic statistical descriptions of the heavy metal concentrations (mg/kg) in roadside soils are listed in Table 3. All of the metal concentrations show a declining trend with the increase of distance to the road edge. The concentrations of metals in meadow soils with high or low sand content are higher than those without sand. Altitude and air pressure do not display a clear pattern associated with the heavy metal content. The heavy metals content is higher along the road segments with asphalt pavement surface than the segments with gravel surface, and they increase with the increase of traffic volume. Comparatively, the concentrations of Zn (116.501 mg/kg), Cd (0.3252 mg/kg) and Pb (44.1208 mg/kg) in roadside soils along the NL road segment are obviously higher than other road segments due to higher traffic volume, and they are also higher than the background values of Zn (74 mg/kg), Cd (0.081 mg/kg) and Pb (29.1 mg/kg) in the Tibet Autonomous Region.

**Table 3:** Concentrations of Cu, Zn, Cd and Pb in roadside soils (mg/kg).

Variable	Class	Cu		Zn		Cd		Pb	
		Mean	S.D.	Mean	S.D.	Mean	S.D.	Mean	S.D.
DIST	0	24.2	5.4	99.6	26.6	0.28	0.18	28.4	19.7
	10	23.1	7.9	97.7	25.1	0.23	0.07	23.0	10.0
	50	20.6	3.2	89.5	13.2	0.21	0.05	20.5	5.5
SOIL	meadow	21.4	3.5	88.5	14.5	0.21	0.05	19.9	8.3
	meals	26.6	10.4	109.9	24.5	0.29	0.21	28.9	14.9
	meahs	24.5	6.0	121.5	37.3	0.31	0.18	45.2	19.7
PRE	560	23.1	2.0	119.6	12.7	0.28	0.02	35.0	12.0
	570	28.7	14.6	97.2	21.0	0.24	0.06	28.4	13.8
	580	22.3	5.1	116.1	32.2	0.25	0.13	39.1	20.1
	590	23.9	4.0	99.4	14.2	0.26	0.12	27.1	11.7
	600	23.8	5.5	104.3	20.7	0.22	0.04	21.8	4.7
	610	21.4	3.5	88.3	18.6	0.23	0.13	19.3	9.4
ALT	4,100	21.4	3.3	85.3	14.3	0.21	0.05	17.6	4.0
	4,200	23.1	7.3	128.2	41.9	0.56	0.46	46.9	29.3
	4,300	20.3	1.2	99.2	1.1	0.16	0.00	20.6	1.1
	4,400	23.8	5.5	104.3	20.7	0.22	0.04	21.8	4.7
	4,500	23.9	4.0	99.4	14.2	0.26	0.12	27.1	11.7
	4,600	21.4	6.0	108.8	23.0	0.23	0.14	42.9	20.1
	4,700	26.4	12.2	106.9	34.4	0.25	0.10	32.6	18.4
	4,800	25.1	7.7	115.1	17.2	0.27	0.05	30.1	10.1
ROAD	ASPHALT	23.4	6.8	100.4	24.7	0.26	0.13	27.0	15.5
	GRAVEL	21.2	3.5	86.5	14.4	0.20	0.05	18.2	3.8
VOLUME	high	23.4	5.9	116.5	32.2	0.33	0.23	44.1	18.2
	middle	22.6	3.6	96.9	13.7	0.22	0.04	20.8	3.6
	low	22.5	6.6	89.0	17.2	0.22	0.05	18.9	6.1
REGION	MQ	22.6	3.6	96.9	13.7	0.22	0.04	20.8	3.6
	NL	23.4	5.9	116.5	32.2	0.33	0.23	44.1	18.2
	NMC	28.7	14.1	105.1	22.2	0.25	0.05	25.9	10.2
	ZE	21.4	3.3	86.1	14.6	0.21	0.05	17.7	4.1

The correlation analysis of the heavy metals’ content in roadside soils indicates that the concentrations of Cu, Zn, Cd and Pb are significantly correlated with each other, as shown in Table 4. The similar conclusions were also obtained from other studies [27,28]. Since there are no industrial factories and farmlands in the study areas, road traffic is the most probable source of these metals [5,29]. The correlation analysis of heavy metals confirms the homology of pollution source for roadside soils.

**Table 4:** Pearson’s correlation between concentrations of Cu, Zn, Cd and Pb.

	Cu	Zn	Cd	Pb
Cu	1	406 (**)	370 (**)	350 (**)
Zn		1	682 (**)	804 (**)
Cd			1	744 (**)
Pb				1

** Correlation is significant at the 0.01 level (2-tailed).

### 3.2. HTBR Modeling and Analyses of Heavy Metal Concentrations

**Figure 2:**
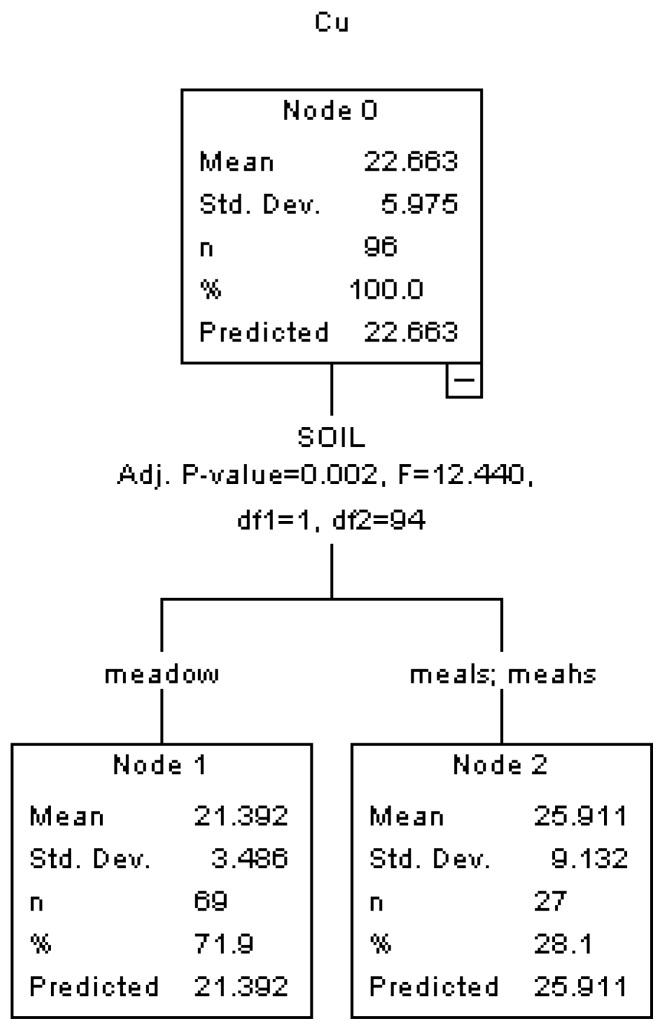
HTBR Model for the concentration of Cu.

Four HTBR models are constructed for predicting and classifying the concentrations of Cu, Zn, Cd and Pb in roadside soils respectively. In the models, the predicting variables include DIST, SOIL, PRE, ALT, ROAD, VOLUME and REGION. The HTBR result for the concentration of Cu is presented in Figure 2. It shows that only SOIL is significantly associated with the concentration of Cu, and the soils with higher content of sand lead to a higher Cu concentration (25.911 mg/kg *vs.* 21.392 mg/kg). Previous studies reported that soil type is an important factor influencing the concentration of Cu [30]. The development of sandy soil is weak [31], and it have larger soil porosity and lower grass density than the pure meadow soils [32], where the deposition is easily stored in the sandy soil pore and the less roadside grasses can intake less heavy metals from the soils by roots [33].

**Figure 3:**
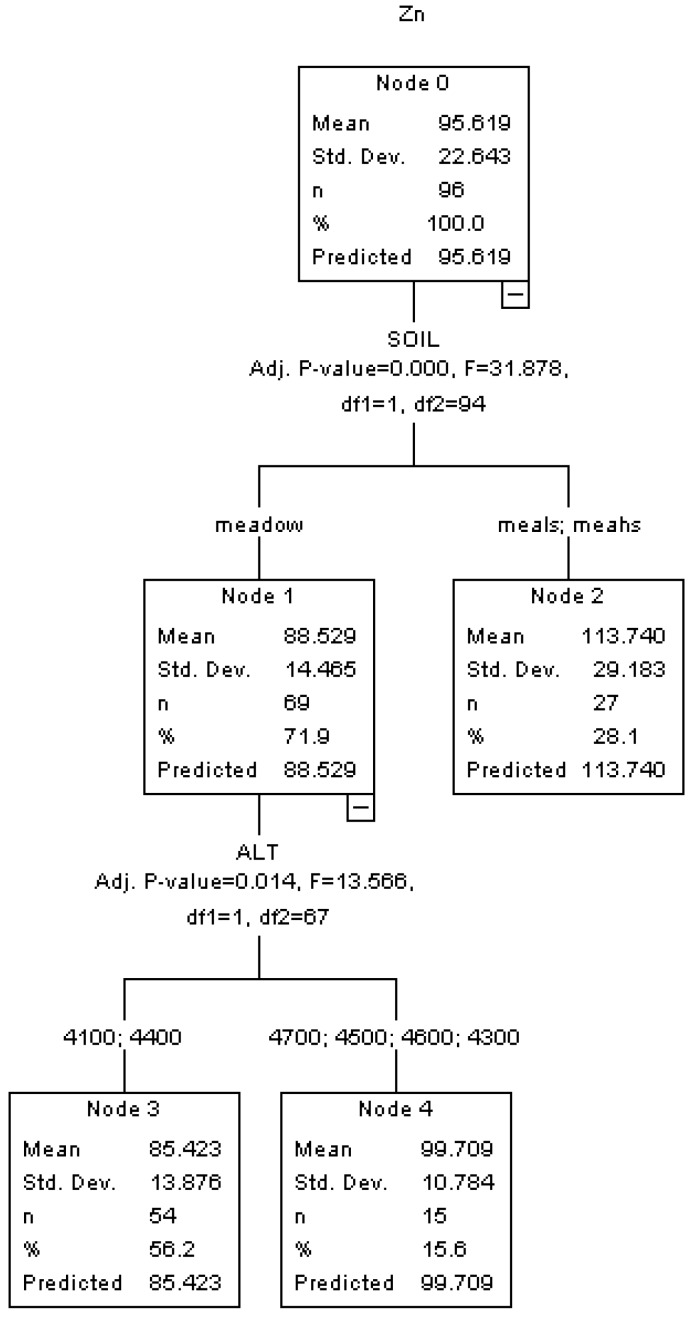
HTBR model for Zn.

As shown in Figure 3, the HTBR result displays that SOIL and ALT are both significantly associated with the concentration of Zn.

Similar to the Cu analysis, the most important factor determining the Zn concentration is soil type (SOIL), and the soils with higher content of sand also result in a higher Zn concentration (113.740 mg/kg *vs.* 88.529 mg/kg). In the second level of the tree, altitude (ALT) leads to the further splits in the pure meadow soil group. The general trend is that the concentration of Zn increases as the altitude increases. The lower altitude group’s Zn concentration is 85.423 mg/kg while the higher altitude group’s Zn concentration is 99.709 mg/kg. This finding is consistent with previous conclusions of heavy metal concentration studies for roadside farmland soil in Nepal [28] and mountain forest soil in China [34]. The percentage of oxygen in the atmosphere decreasing with the increment of altitude can influence the efficiency of gas consumption and vehicular emission mechanism. Previous research focusing on the effect of altitude on vehicle on-road emissions indicated that vehicular emissions at high altitude can be much higher than observed at sea level [35].

**Figure 4:**
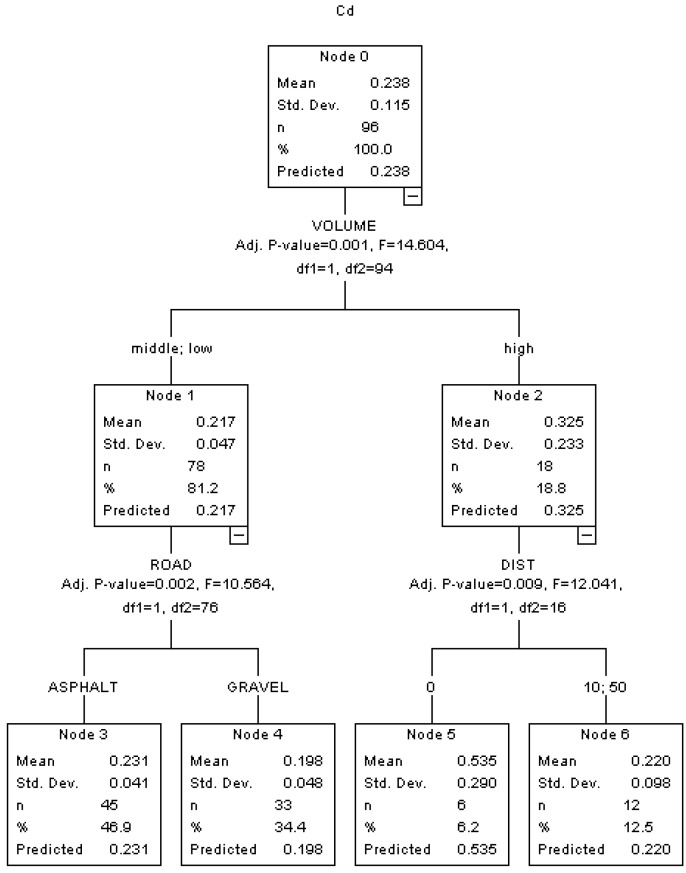
HTBR model for Cd.

In the HTBR Model as shown in Figure 4, VOLUME, ROAD and DIST are significant variables determining the concentration of Cd. The top of the tree shows that traffic volume (VOLUME) is the most important factor. 

The Cd concentration in roadside soils with high traffic volume is higher than those with middle and low traffic volume (0.325 mg/kg *vs.* 0.231 mg/kg). Engine oil consumption is responsible for the largest emission for Cd [36], which is directly dependent on the number of vehicles traveling in the road segments. In the high traffic volume group, the Cd concentration is further split by roadside distance (DIST). The mean concentration of Cd in the soils with 0 m roadside distance is more than twice higher than that with 10 m or 50 m roadside distances (0.535 mg/kg *vs.* 0.220 mg/kg). Many studies have reported that heavy metal concentrations are positively related to traffic volume [5,6]. A recent observational study indicated that the traffic-related heavy metals accumulation in roadside soils decrease with distance to the road only in old roads that have undergone the traffic impact for a long time, but not in new roads or roads with low traffic density [37].

**Figure 5:**
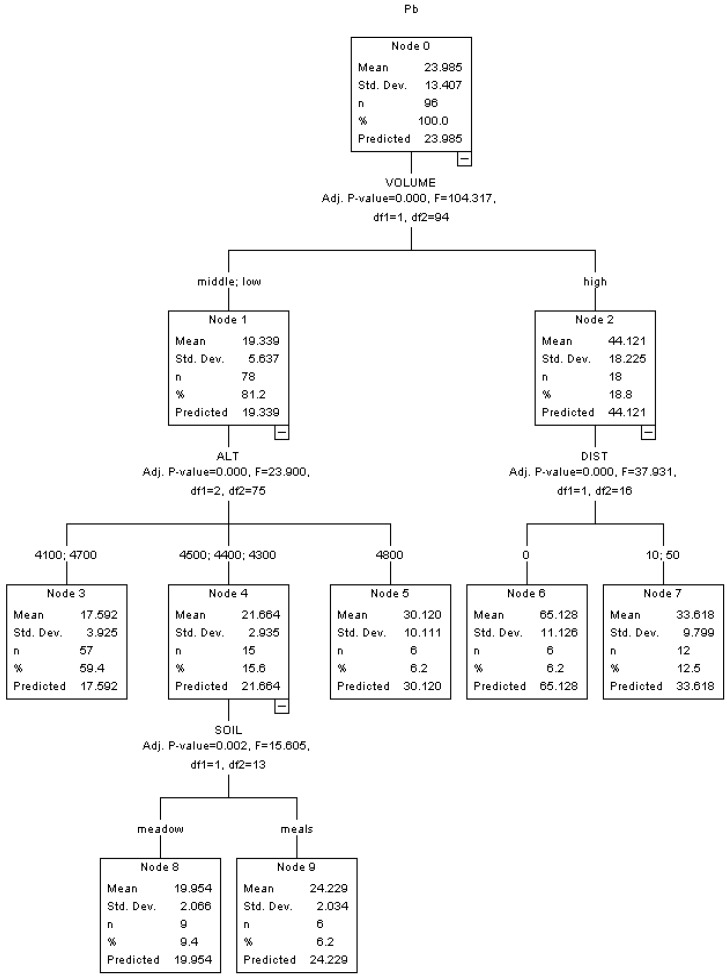
HTBR model for Pb.

Normally, the heavy metal content in roadside soils has a belt-shaped distribution in terms of distance to road edge, decreasing exponentially with distance from road [38]. In the low and middle traffic volume group, the Cd concentration is further split by road surface type (ROAD). The mean concentration of Cd in the soils along the road segments with asphalt surface is higher than that along the road segments with gravel surface (0.231 mg/kg *vs.* 0.198 mg/kg). Bitumen and mineral filler materials in the asphalt road surfaces contain different heavy metal species, including Cu, Zn, Cd and Pb [39]. Thus, the asphalt road surface erosion may contribute to more emission for Cd into roadside soil.

In the HTBR Model as shown in Figure 5, VOLUME, ALT, DIST and SOIL are significant variables classifying the concentration of Pb. Similar to Cd, the top of the tree shows that traffic volume (VOLUME) is the most important factor to determine the concentration of Pb. The Pb concentration in roadside soils with high traffic volume is more than twice higher than that with middle and low traffic volume (44.121 mg/kg *vs.* 19.339 mg/kg). Brake wear and fuel emissions are the most important sources of Pb emissions [38], both of which are dependent on the level of traffic volume. In the second level of the tree, altitude and roadside distance lead to further splits. For the low and middle traffic volume group, the concentration of Pb is split into three subgroups by altitude: 4,100 m or 4,700 m (17.592 mg/kg), 4,300 m–4,500 m (21.664 mg/kg) and 4800 m (30.120 mg/kg). Similar to Zn, the general trend is that the concentration of Pb increases as the altitude increases, except for 4,700 m. For the high traffic volume group, the concentration of Pb is split by roadside distance: 0 m subgroup (65.128 mg/kg) and 10 m–50 m subgroup (33.618 mg/kg), which are significantly higher than the background values in both Qinghai Province (20.9 mg/kg) and Tibet Autonomous Region (29.1 mg/kg). In the third level of the tree, soil type lead to the further split of 4,300 m–4,500 m altitude group. Similar to Cu and Zn, the soils with low content of sand has a higher Pb concentration than the pure meadow soils (24.229 mg/kg *vs.* 19.954 mg/kg). 

### 3.3. Assessment of Heavy Metals Contamination

**Figure 6:**
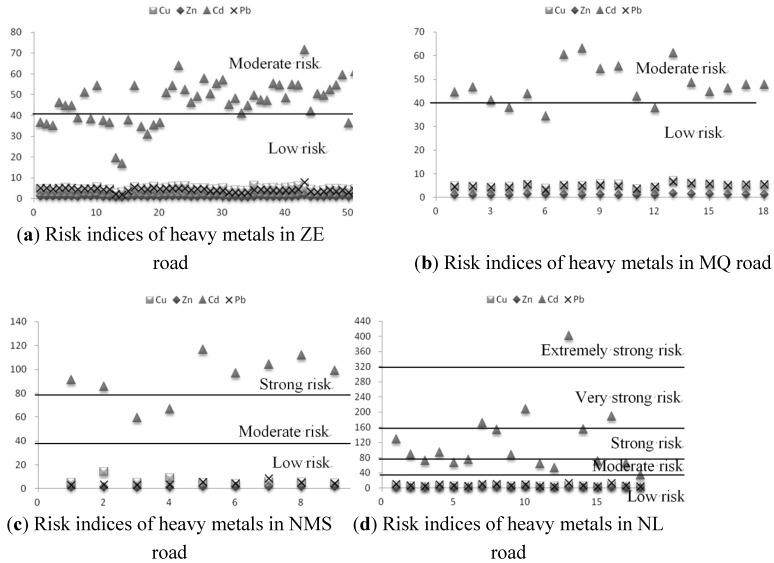
Hakanson potential ecological risk indices of heavy metals for the four road segments.

The four road segments monitored in this study are located in different regions: ZE and NMS road segments are connected to the plateau lakes, and MQ and NL road segments are nearby plateau lakes. The potential ecological risk indices of Cu, Zn, Cd and Pb in each region are listed in Table 5, which includes parameters used to calculate the potential ecological risk indices. We define the total value as the comprehensive potential ecological risk index of one region, which is the sum of four metals’ values (Ei r) in a region. In the four regions, all of the indices of Cu, Zn and Pb accumulations are far below 40, indicating a safe level. However, the Ei r values of Cd in the four regions reach higher levels. The Cd indices in ZE and MQ regions are 46.37 and 47.62 respectively, indicating a moderate potential ecological risk. The Cd indices in NMS and NL regions are as high as 92.02 and 120.43 respectively, indicating a strong potential ecological risk level. Further, the potential ecological risk indices of four metals for each sampling site are presented in Figure 6. It shows that the roadside soils along the four road segments have a potential ecological risk of Cd contamination at different degrees: about 82% of ZE road segment samples and 83% of MQ road segment samples are at a moderate potential ecological risk level; For NMS road segment, 33% of samples are at a moderate potential ecological risk level, and 67% of samples are at a strong potential ecological risk level; For NL road segment, 39% of samples are at a moderate potential ecological risk level, 33% of the samples are at a strong potential ecological risk level, 17% of the samples are at a very strong risk level, and even 6% of the samples are at an extremely strong risk level. In Figure 6, the individual sampling site risk analyses indicate that all of the accumulations of Cu, Zn and Pb in roadside soils remain a safe level but some spots with peak concentrations have been severely polluted by Cd due to traffic activities. As indicated in the HTBR analysis, traffic volume is the most important factor influencing the Cd concentration in roadside soil. Therefore, the order of road traffic volume (ZE < NMS < MQ < NL) results in the same order of Cd risk index, as well as the total risk index.

**Table 5:** The potential ecological risk indices of Cu, Zn, Cd and Pb in each region.

Region	Elements	Ci s	Ci n	Ci f	Ti r	Ei r	Total
ZE	Cu	21.39	22.20	0.96	5	4.82	56.49
	Zn	86.13	80.30	1.07	1	1.07	
	Cd	0.21	0.14	1.55	30	46.37	
	Pb	17.67	20.90	0.85	5	4.23	
MQ	Cu	22.56	22.20	1.02	5	5.08	58.87
	Zn	96.87	80.30	1.21	1	1.21	
	Cd	0.22	0.14	1.59	30	47.62	
	Pb	20.77	20.90	0.99	5	4.97	
NMS	Cu	28.70	21.90	1.31	5	6.55	104.45
	Zn	105.13	74.00	1.42	1	1.42	
	Cd	0.25	0.08	3.07	30	92.02	
	Pb	25.95	29.10	0.89	5	4.46	
NL	Cu	23.35	21.90	1.07	5	5.33	134.92
	Zn	116.50	74.00	1.57	1	1.57	
	Cd	0.33	0.08	4.01	30	120.43	
	Pb	44.12	29.10	1.52	5	7.58	

## 4. Conclusions

Heavy metal concentrations in roadside soils are not a new research topic and have been studied in many countries and regions. This research investigated the four typical heavy metals content (Cu, Zn, Cd and Pb) in roadside soils nearby three lakes and in adjacent regions on the Tibetan Plateau. This study can still contribute to the research literature from the following aspects. First, the sampling areas are far from agricultural and industrial activities, so the extra heavy metal accumulations in roadside soils are almost purely due to traffic activities, which is helpful to clarify the mechanism of traffic pollution and associated influencing factors. 

Secondly, no previous studies have applied the HTBR method to explain heavy metal concentration patterns. This study displays that the HTBR models can effectively identify the important factors associated with heavy metal concentrations for both analysis and predictive purposes. It was found that soil type is significantly associated with the concentrations of Cu, Zn and Pb and the Tibetan Plateau meadow soils with higher content of sand lead to higher concentrations of the heavy metals. The most important factor to determine the concentrations of Cd and Pb is traffic volume: the higher the traffic volume of road segments, the higher the heavy metals concentrations in roadside soils. For the high traffic volume, the Cd and Pb concentrations decrease significantly with the roadside distance. An interesting finding of this study is that the concentrations of Zn and Pb increase as the altitude of sampling site increases, presumably because that the lower percentage of oxygen in the atmosphere at the higher altitude sites can degrade the efficiency of gas consumption and increase vehicular emissions.

Finally, this research focuses on the road segments adjacent to plateau lakes. Dynamically monitoring and assessing heavy metals contamination of roadside soils in the regions close to the plateau lake is important for aquatic ecosystem health security. Since the potential ecological risk indices of Cd in the sampling regions indicate a strong risk level for NMS and NL regions, it is suggested to further investigate the Cd content in lake water, lake shores and lake sediments for a sustainable development.
